# The Cerebellar Dopaminergic System

**DOI:** 10.3389/fnsys.2021.650614

**Published:** 2021-08-05

**Authors:** Paolo Flace, Paolo Livrea, Gianpaolo Antonio Basile, Diana Galletta, Antonella Bizzoca, Gianfranco Gennarini, Salvatore Bertino, Jacopo Junio Valerio Branca, Massimo Gulisano, Simona Bianconi, Alessia Bramanti, Giuseppe Anastasi

**Affiliations:** ^1^Medical School, University of Bari ‘Aldo Moro', Bari, Italy; ^2^University of Bari “Aldo Moro”, Bari, Italy; ^3^Department of Biomedical, Dental Sciences and Morphological and Functional Images, University of Messina, Messina, Italy; ^4^Unit of Psychiatry and Psychology, Federico II University Hospital, Naples, Italy; ^5^Department of Basic Medical Sciences, Neuroscience and Sense Organs, University of Bari “Aldo Moro”, Bari, Italy; ^6^Department of Experimental and Clinical Medicine, University of Firenze, Firenze, Italy; ^7^Physical, Rehabilitation Medicine and Sport Medicine Unit, University Hospital “G. Martino”, Messina, Italy; ^8^Scientific Institute for Research, Hospitalization and Health Care IRCCS “Centro Neurolesi Bonino Pulejo”, Messina, Italy

**Keywords:** cerebellum, dopamine, dopamine receptors, non-traditional large neurons, Parkinson's disease, schizophrenia, autism spectrum disorders

## Abstract

In the central nervous system (CNS), dopamine (DA) is involved in motor and cognitive functions. Although the cerebellum is not been considered an elective dopaminergic region, studies attributed to it a critical role in dopamine deficit-related neurological and psychiatric disorders [e.g., Parkinson's disease (PD) and schizophrenia (SCZ)]. Data on the cerebellar dopaminergic neuronal system are still lacking. Nevertheless, biochemical studies detected in the mammalians cerebellum high dopamine levels, while chemical neuroanatomy studies revealed the presence of midbrain dopaminergic afferents to the cerebellum as well as wide distribution of the dopaminergic receptor subtypes (DRD_1_-DRD_5_). The present review summarizes the data on the cerebellar dopaminergic system including its involvement in associative and projective circuits. Furthermore, this study also briefly discusses the role of the cerebellar dopaminergic system in some neurologic and psychiatric disorders and suggests its potential involvement as a target in pharmacologic and non-pharmacologic treatments.

## Introduction

In the mammalian cerebellum, the neurotransmitter systems traditionally involved in the synaptic and extrasynaptic interactions may include the excitatory glutamatergic system (Clements et al., 1987; Batini et al., 1992; Ottersen, 1993; Zhang and Ottersen, 1993; Batchelor et al., 1994; Grandes et al., 1994; Nusser and Somogyi, 1997; Knöpfel and Grandes, 2002; Hioki et al., 2003; Sanchez-Perez et al., 2005; Benagiano et al., 2011; Mugnaini et al., 2011; Uusisaari and De Schutter, 2011; Mapelli et al., 2015) as well as the inhibitory GABAergic and glycinergic systems (Gabbott et al., 1986; Wuenschell et al., 1986; Batini et al., 1992; Ottersen, 1993; Wisden et al., 1996; Sastry et al., 1997; Benagiano et al., 2000a,b; Flace et al., 2004; Crook et al., 2006; Tabata and Kano, 2006; Uusisaari and De Schutter, 2011; Mapelli et al., 2015), which are both involved in intrinsic and projective cerebellar circuits (Fredette and Mugnaini, 1991; Uusisaari and De Schutter, 2011; Ankri et al., 2015; Mapelli et al., 2015; Gao et al., 2016). Moreover, in several studies, the existence of a cerebellar cholinergic system (Jaarsma et al., 1997; Prestori et al., 2013; Zhang et al., 2016) and several neuropeptidergic systems have been demonstrated (King et al., 1992; Joo et al., 2004; Schibusawa et al., 2008; Benagiano et al., 2009; Ito, 2009). Currently, data on the presence and distribution of monoaminergic systems in the mammalian cerebellum are still incomplete and not fully analyzed.

Studies reported in the developmental and adult mammalian cerebellum the presence of extrinsic monoaminergic pathways. Studies have been mainly focused on the cerebellar functional role of serotonin (5-HT) and noradrenaline (NA); as a result, until now, the functional role of dopamine (DA) in the cerebellum has been widely disregarded.

In studies using histofluorescence (Hökfelt and Fuxe, 1969) or immunohistochemical methods through specific 5-HT antiserum, in several mammals, including humans, the presence of a cerebellar serotonergic fiber system (Takeuchi et al., 1982; Kerr and Bishop, 1991; Ottersen, 1993; Kitzman and Bishop, 1997; Flace, 2017, 2019a), composed by 5-HT immunoreactive axonal plexuses of fibers and by neuronal cell bodies and processes distributed in the cerebellar cortical layers and in the deep cerebellar nuclei, has been demonstrated (Takeuchi et al., 1982; Bishop and Ho, 1985; Kerr and Bishop, 1991; Crivellato et al., 1992; Flace, 2017, 2019a).

The cerebellar serotonergic fibers originate mostly by the serotonergic cell groups of the reticular formation (B_1_-B_3_, B_6_, B_7_, and B_9_; Dahlström and Fuxe, 1964; Bishop and Ho, 1985; Törk, 1990, Kerr and Bishop, 1991; Kitzman and Bishop, 1994, 1997). In the cerebellar cortex and the deep cerebellar nuclei, different serotonergic subtype receptors such as 5-HT_1B_, 5-HT_2A_, 5-HT_2B_, 5-HT_3_, and 5-HT_5A_ have been demonstrated (Duxon et al., 1997; Pasqualetti et al., 1998; Sari et al., 1999; Geurts et al., 2002; Oostland et al., 2013; Marinova et al., 2015). During the development, a role of 5-HT in dendritic growth and synaptic plasticity mechanisms has been demonstrated (Bishop et al., 1988; Oostland and van Hooft, 2013; Oostland et al., 2013).

In the adult cerebellum, 5-HT play a role in the modulation of the GABAergic and glutamatergic signaling (Strahlendorf et al., 1991; Cumming-Hood et al., 1993; Kitzman and Bishop, 1997; Dieudonné and Dumoulin, 2000; Di Mauro et al., 2003; Saitow et al., 2009; Murano et al., 2011). 5-HT decreases the activity of the Purkinje neurons (Kerr and Bishop, 1992) by means of the serotonergic receptor 5-HT1_A_ (Mitoma and Konishi, 1996, 1999). 5-HT may set PCs at a preferred firing rate by modulation of transient outward h currents (Strahlendorf et al., 1984; Wang et al., 1992).

5-HT is involved in the long-term cerebellar effects, as the modulation of postsynaptic induction of long term depression (LTD), mainly by means of the serotonergic receptors 5-HT_2A_ and 5-HT_2B_, which have been expressed on the Purkinje neurons (Maeshima et al., 1998; Cornea-Hébert et al., 1999).

In addition, these serotonergic receptor subtypes activate phospholipase C, resulting in the production of inositol-3 trisphosphate (IP3), which can regulate the threshold of regenerative cycles of Ca^2+^ elevation (Raymond et al., 2001). In chemical neuroanatomy studies, the presence of noradrenergic innervation in the cerebellum of rodents and primates (including humans; Hökfelt and Fuxe, 1969; Siggins et al., 1971; Landis and Bloom, 1975; Yamamoto et al., 1977; Pasquier et al., 1980; Hayashi, 1987; Pompeiano et al., 1989; Powers et al., 1989; Yew et al., 1995; Rosin et al., 1996; Talley et al., 1996; Gould et al., 1997; Melchitzky and Lewis, 2000) by means of fluorescent histochemistry (Falck and Torp, 1962; Hökfelt and Fuxe, 1969), or by specific antisera for dopamine β hydroxylase (DBH), the NA biosynthesizing enzymes has been demonstrated (Fritschy and Grzanna, 1989). Cerebellar noradrenergic fibers mainly originate from the noradrenergic cell groups of the reticular formation (A_4_-A_7_; Dahlström and Fuxe, 1964; Hökfelt and Fuxe, 1969; Pickel et al., 1973; Pasquier et al., 1980; Dietrichs, 1988; Powers et al., 1989). Such noradrenergic fibers are localized in the three cerebellar cortical layers and in the deep cerebellar nuclei, oriented so as to generate axonal plexuses (Sachs et al., 1973; Pasquier et al., 1980; Dietrichs, 1985; Felten et al., 1986; Powers et al., 1989; Melchitzky and Lewis, 2000).

In the human developmental cerebellum, at 16–18 and 26–28 weeks, a transient expression of noradrenergic neuronal cell bodies and processes occurs in the cerebellar cortex and in the deep cerebellar nuclei has been demonstrated (Yew et al., 1995). In addition, in the cerebellum of mammals, extensive distribution of the β_2_ adrenergic subtype receptor (Pompeiano et al., 1989; Voogd et al., 1996) and, to a lesser extent, of β_1_, α_1_, and α_2_ adrenergic subtype receptors have been demonstrated (Pompeiano et al., 1989; McCune et al., 1993; Rosin et al., 1996; Talley et al., 1996; Voogd et al., 1996). In the development, it has been found that the cerebellar noradrenergic system influences mainly the GABAergic synaptogenesis (Sievers et al., 1981; Sievers and Klemm, 1982; O'Leary and Leslie, 2003; Happe et al., 2004; Hirono et al., 2014). In the adult cerebellum, NA plays a pivotal role in the modulation of the glutamatergic and GABAergic synaptic signaling (Moises et al., 1983; Woodward et al., 1991; Hirono and Obata, 2006; Hirono et al., 2014; Lippiello et al., 2015). Noradrenaline exerts on the Purkinje neurons two types of influence. An increase of the intracellular levels of cAMP protein kinase-dependent by means on the beta-adrenergic receptor (Kano et al., 1992; Cheun and Yeh, 1996); the levels of cAMP can, in turn, enhance a form of neuronal plasticity called rebound potentiation (RP; Kano et al., 1992; Cheun and Yeh, 1996; Kawaguchi and Hirano, 2002). Moreover, NA influences in the Purkinje neurons the expression of the immediate-early genes, c-fos and Jun-B (Pompeiano, 1998). The induction of immediate-early genes in the Purkinje neurons appears to play a role in the long-term biochemical changes involved in the maintenance of cerebellar long-term plasticity such as LTD (Pompeiano, 1998).

On the other hand, currently, the presence and the distribution of a dopaminergic system in the cerebellum and its functional role is controversial or neglected (Oertel, 1993; Ottersen, 1993; Kwong et al., 2000). However, several studies demonstrated the involvement of the cerebellum in DA related neurological and psychiatric disorders, such as Parkinson's disease (PD), schizophrenia (SCZ), autism spectrum disorders (ASD), and drug addiction (Glaser et al., 2006; Andreasen and Pierson, 2008; Mittleman et al., 2008; O'Hallaran et al., 2012; Lewis et al., 2013; Wu and Hallett, 2013; Parker et al., 2014, Carta et al., 2019; Gil-Miravet et al., 2019; Miquel et al., 2020). Therefore, the goal of the present review is to provide a comprehensive overview of the presence, distribution, and functional role of the cerebellar dopaminergic system, also discussing its potential pathophysiological and clinical implications in some neurological and psychiatric DA-related disorders.

## Morphological Aspects of the Dopaminergic Cerebellar System

Although the presence of a dopaminergic system in the cerebellum is in part predictable, currently, the cerebellum is not strictly considered a dopaminergic area (Glowinski and Iversen, 1966; Lindvall and Björklund, 1974; Beckstead et al., 1979; Ottersen, 1993; Masilamoni et al., 2010). In biochemical studies, high levels of DA in the human postmortem cerebellum (Adolfsson et al., 1979; Roubein and Embree, 1979; Spokes, 1979; Gottfries, 1980) and in the rat and monkey cerebellum were detected (Versteeg et al., 1976; Mefford et al., 1982; Glaser et al., 2006; Quansah et al., 2018). Furthermore, in the mammalian cerebellum, *in vivo* studies by means of positron emission tomography (PET) revealed a significant presence of selective dopamine transporter ligands (DAT-Ls) (Schoeps et al., 1993; Lundkvist et al., 1995; Hall et al., 1999; Emond et al., 2008; Varrone et al., 2009; Jiang et al., 2019).

Chemical neuroanatomy studies on the detection of dopaminergic neuronal elements in the cerebellum of mammals (including human) makes use of direct antisera against DA and of [^3^H]-dopaminergic ligands (Panagopoulos et al., 1991; Panagopoulos and Matsokis, 1994) or antisera against the specific dopaminergic marker, the dopamine transporter (DAT), the plasma membrane monoamine transporter involved in DA synaptic reuptake (Table 1; Melchitzky and Lewis, 2000; Dunnet et al., 2005; Giompres and Delis, 2005; Delis et al., 2008; Kim et al., 2009; Flace et al., 2019b, 2020), the indirect marker of the dopaminergic neurotransmission, the dopamine and adenosine 3′-5′-monophosphate (cAMP)-regulated protein Mr 32,0000 (DARPP-32), a protein phosphatase-1 inhibitor involved in dopaminergic neuronal synaptic signaling (Table 1; Alder and Barbas, 1995; López et al., 2010; Nishi and Shuto, 2017), or, indirectly, by means of antisera against not elective markers for DA, such as tyrosine hydroxylase (TH), the rate-limiting enzyme DA biosynthesis, which catalyzes the conversion of L-tyrosine to L-3,4-dihydroxyphenylalanine (L-DOPA) (Table 1; Ikai et al., 1992; Fujii et al., 1994; Melchitzky and Lewis, 2000; White and Thomas, 2012) and vesicular monoamine transporter 2 (VMAT_2_), the synaptic vesicles transporter of monoamine neurotransmitters such as DA, NA, 5-HT, and histamine (HIS) (Table 1; Kim et al., 2009; Lawal and Krantz, 2013).

**Table 1:** Distribution of the catecholaminergic and dopaminergic markers in the mammalian cerebellum.

**Catecholaminergic and dopaminergic marker**	**Molecular layer**	**Purkinje neuron layer**	**Granular layer**	**Deep cerebellar nuclei**	**Cerebellar lobules Larsell, 1952**
Tyrosine hydroxylase (TH) (catecholaminergic marker)	- Fibers climbing-like oriented - Fibers in the neuropil	- Purkinje neurons cell bodies and processes (lobulesVI-X), - Fibers around Purkinje neuron cell bodies	- Fibers in the neuropil - Mossy fiber rosettes-like	- Fibers in the neuropil of all nuclei	Lobules I, III, V, VI, VIII, IX, X, Crus I, Crus II, paraflocculus
Vesicular Monoamine Transporter 2 (VMAT_2_) - (catecholaminergic marker)	Axon terminals (puncta) around dendrites of Purkinje neurons	Axon terminals (puncta) around Purkinje neuron cell bodies	–	–	Lobule IX B
Dopamine Transporter (DAT) (dopaminergic marker)	- Fibers in the neuropil - Dendrites of Purkinje neurons	- Purkinje neurons cell bodies and processes - Fibers in the neuropil	- Fibers randomly distributed - Clusters in the sites of glomeruli complex - Granules cell bodies (occasionally) - Cell bodies and processes of Synarmotic neurons and of perivascular neurons	- Fibers and puncta (axon terminals) - Cell bodies and processes of projective and associative neurons in all nuclei	All lobules, (lobules VII, IX in human)
Dopamine and Adenosine 3′-5′-monophosphate (cAMP) Regulated Protein Mr 32,000 (DARPP-32) (indirect dopaminergic marker)	Dendrites of Purkinje neurons	Purkinje neurons cell bodies and processes	–	–	All lobules

During the development of the mouse cerebellar cortex, a transient expression of TH in Purkinje neurons in different ages from postnatal day 3 (P3) to 11 months (M11) has been observed (Fujii et al., 1994). The TH expression appears in the Purkinje neurons at P8 in the cerebellar vermis, increases at P13–P15, reduces at P19, and then increases again after 1 month of age, reaching a maximum expression at 11 months (Fujii et al., 1994).

In the adult mouse cerebellum, the TH immunoreactive fibers are in the vermal lobules V and VI, whereas the lowest numbers are located in lobule X, and in each deep cerebellar nuclei, a dense plexus of TH immunoreactive varicose fibers has been mainly detected (Table 1; Nelson et al., 1997). Whereas, TH immunoreactive cell bodies of Purkinje neurons have been found in the flocculus, paraflocculus, vermal lobules VI–X, and in the hemispheric lobules IX–X (Table 1; Nelson et al., 1997). In pharmacological studies, in the mouse cerebellum DA specific binding sites of [^3^H]DA and [^3^H]spiperone has been detected (Panagopoulos and Matsokis, 1994).

Moreover, in the adult mouse cerebellum, specific binding of the DA uptake inhibitor ^3^[H]GBR12935 in the paraflocculus, lobules IV, VI, IX, X, and lobule simplex Crus I and II has been detected (Delis et al., 2008). In the cerebellar cortex, the specific binding of ^3^[H]GBR12935 was mainly distributed in the molecular layer and in the granular layer, while DAT immunoreactivity has been mainly detected in the cell bodies of the Purkinje neurons and in some neuron types of the deep cerebellar nuclei (Table 1; Delis et al., 2008). Furthermore, in the mouse cerebellum, DARPP-32 immunoreactive Purkinje neuron cell bodies in the laminae of all lobules have been observed (Table 1; Alder and Barbas, 1995).

In the rat cerebellum, the DA immunoreactivity presents a uniform distribution pattern in all lobules, and in the layers of the cerebellar cortex the DA immunoreactivity was mainly detected in the molecular layer in climbing fiber-like forms, while a small number of DA immunoreactive fibers within the Purkinje neuron layer and in the granular layer were found (Table 1; Panagopoulos et al., 1991).

Furthermore, in the rat cerebellum, the TH immunoreactive fibers in the paraflocculus and crus I and II ansiform lobules have been mainly detected (Table 1; Ikai et al., 1992); whereas, a high number of VMAT_2_ immunoreactive ‘puncta' (attributable to axon terminals or short sections of dendrites) has been observed in the lobule IX of the posterior cerebellum (Table 1; Kim et al., 2009).

In the rat cerebellar cortex, a low number TH immunoreactive fibers variously oriented in the Purkinje neuron layer and in the granular layer has been detected; instead, in the molecular layer, a high number of climbing-like oriented TH immunoreactive fibers has been observed (Table 1; Takada et al., 1993), and VMAT_2_ small immunoreactive “puncta” were observed between the Purkinje neuron cell bodies and in the molecular layer in close relationship with the dendritic arborizations of the Purkinje neurons (Table 1; Kim et al., 2009).

Biochemical analysis revealed significant levels of DA in the deep cerebellar nuclei of rat, with the highest DA levels being localized in the fastigial and dentate nuclei (Glaser et al., 2006), which is in line with a morphological study that revealed the presence of DAT immunoreactive fibers in all deep cerebellar nuclei (Delis et al., 2008). Moreover, a wide distribution of DAT immunoreactive fibers in the three layers of the cerebellar cortex and in the deep cerebellar nuclei has been revealed (Delis et al., 2008).

In addition, DARPP-32 immunoreactive dendritic arborization of the Purkinje neurons in the molecular layer of all cerebellar lobules has been observed (Table 1; Alder and Barbas, 1995).

In the rat cerebellar cortex, the presence of TH immunoreactive cell bodies of Purkinje neurons has been demonstrated in the lobules I and X of the vermis, in the paraflocculus, and in crus I and II ansiform lobules (Table 1; Takada et al., 1993) Instead, Kim et al. (2009) evidenced the presence of TH immunoreactive cell bodies of Purkinje neurons predominantly in the lobules VIII–X and a discontinuous presence in the lobules VI and VII, whereas a high number of DAT immunoreactive cell bodies of Purkinje neurons has been detected in the lobule IX of the posterior cerebellum (Table 1; Kim et al., 2009).

In the opossum cerebellum, most of TH immunoreactive fibers have been found in the lobules III-VIII of the vermis and, to a lesser extent, in lobules I and X (Table 1; Nelson et al., 1997). Moreover, in the opossum cerebellar cortex, the TH immunoreactive fibers were mainly localized in the Purkinje neurons layer; they surround the cell bodies of the Purkinje neurons or run parallel to the plane of the Purkinje neuron layer, whereas, in the molecular layer, only a small amount was detected (Table 1; Nelson et al., 1997), and in the granular layer, the TH immunoreactive fibers featured a random distribution (Nelson et al., 1997).

In addition, a moderate number of randomly distributed TH immunoreactive fibers has been detected in the deep cerebellar nuclei (Nelson et al., 1997).

In the cat cerebellum, the highest density of TH immunoreactive fibers were distributed in the vermal lobules V and VI and in the hemispheric lobules VI and crus I and II; meanwhile, the lowest density of fibers has been observed in lobules I–III and VIII–X (Table 1; Nelson et al., 1997). In the cat cerebellar cortex, the high density of TH immunoreactive fibers have been observed in the granular layer, where they present a random orientation, and in the Purkinje neurons layer, where they surround the cell bodies of the Purkinje neurons have been observed. Instead, in the molecular layer, only a few densities of TH immunoreactive fibers with a perpendicular orientation that often extend radially to the surface of the pial surface of the cortex have been detected (Table 1; Nelson et al., 1997). Finally, a moderate density of TH immunoreactive varicose fibers in the deep cerebellar nuclei have been also found (Table 1; Nelson et al., 1997).

In the monkey cerebellum, we found a low density of TH immunoreactive fibers to be distributed in the lobules of the vermis and of both cerebellar hemispheres, whereas the DAT immunoreactive fibers were only observed in the vermis of the following lobules II, III, IV, VIIIA, VIIIB, IX, and X (Table 1; Melchitzky and Lewis, 2000).

In the monkey cerebellar cortex, a low density of TH immunoreactive fibers in the granular layer and in the molecular layer, has been detected while a higher density of TH plexuses and axonal terminals just beneath the Purkinje neuron cell bodies has been found. Conversely, DAT immunoreactive fibers to be randomly distributed in the granular layer; however, we also found forming plexuses around the deep pole of the cell bodies of the immunonegative Purkinje neurons has been observed. In contrast, in the molecular layer, no DAT immunoreactive fibers have been detected (Table 1; Melchitzky and Lewis, 2000).

Currently, in the monkey cerebellum studies, there is no evidence that proves the existence of dopaminergic neurons. Despite this, a biochemical study demonstrated significant levels of DA in all deep cerebellar nuclei, and the highest levels have been detected in the interpositus and dentate nuclei, (Glaser et al., 2006). Furthermore, in all cerebellar lobules of the monkey cerebellar cortex, a wide presence of DARPP-32 immunoreactive cell bodies and dendritic arborizations of Purkinje neurons has been detected (Table 1; Alder and Barbas, 1995).

**Figure 1:**
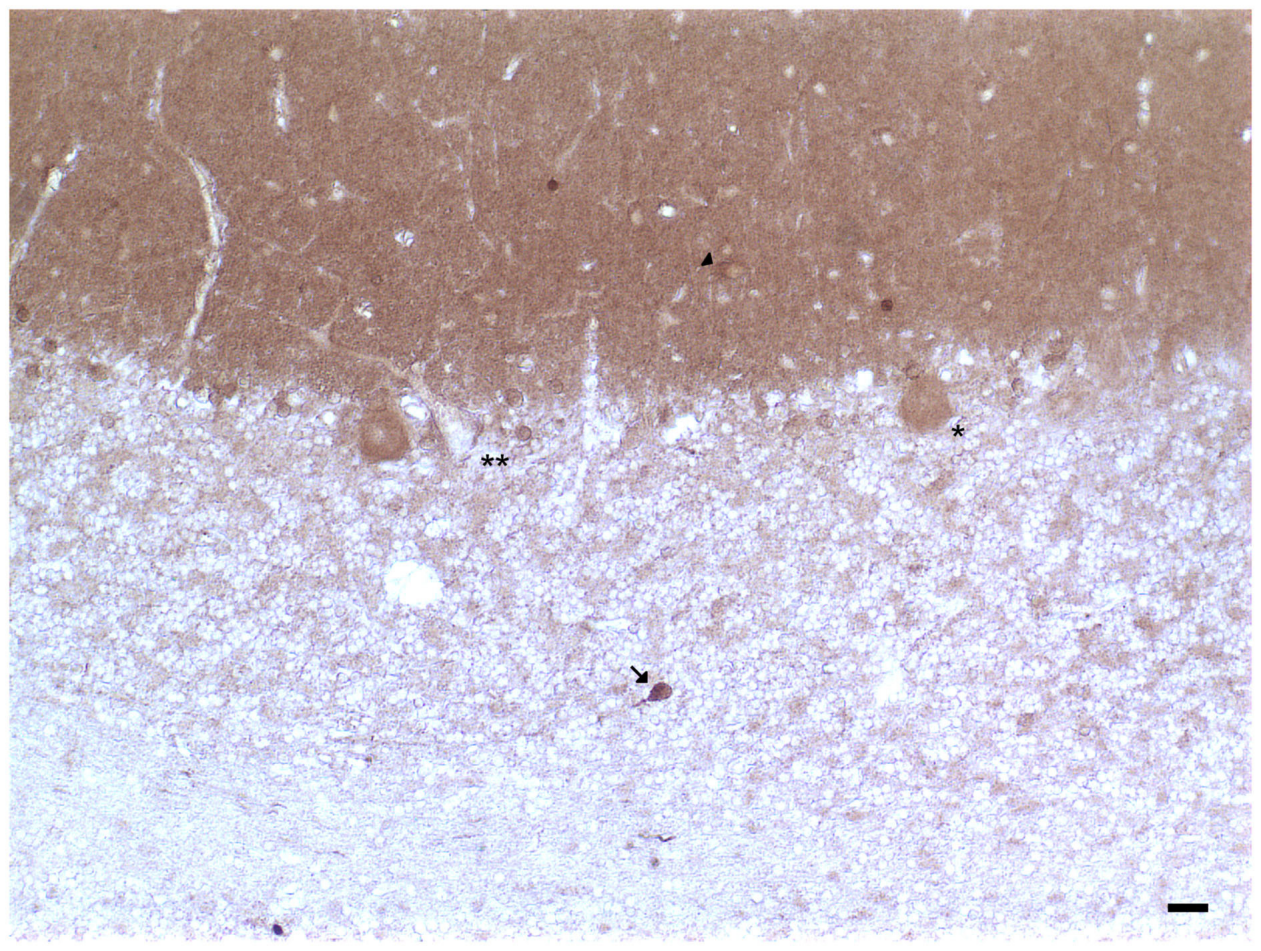
Dopamine transporter (DAT) immunoreactivity in the cerebellar cortex. The DAT immunoreactivity is detectable in neuronal bodies and processes of all the layers of the cerebellar cortex. In the molecular layer, DAT immunoreactivity in basket neurons (*arrowheads*); primary and secondary trunks dendritic and apical dendrites of Purkinje neurons; immunonegative stellate neurons, fine clusters of DAT immunoreactivity in the neuropil of the layer. In the Purkinje neuron layer, DAT immunoreactive Purkinje neuron cell body (*single asterisk*), DAT immunonegative Purkinje neuron (*double asterisk*). In the granular layer, DAT immunoreactivity in space of Held, DAT immunoreactivity in the cell body, and axon-like processes of the synarmotic neuron (*arrow*). (Scale bar: 25 μm).

**Figure 2:**
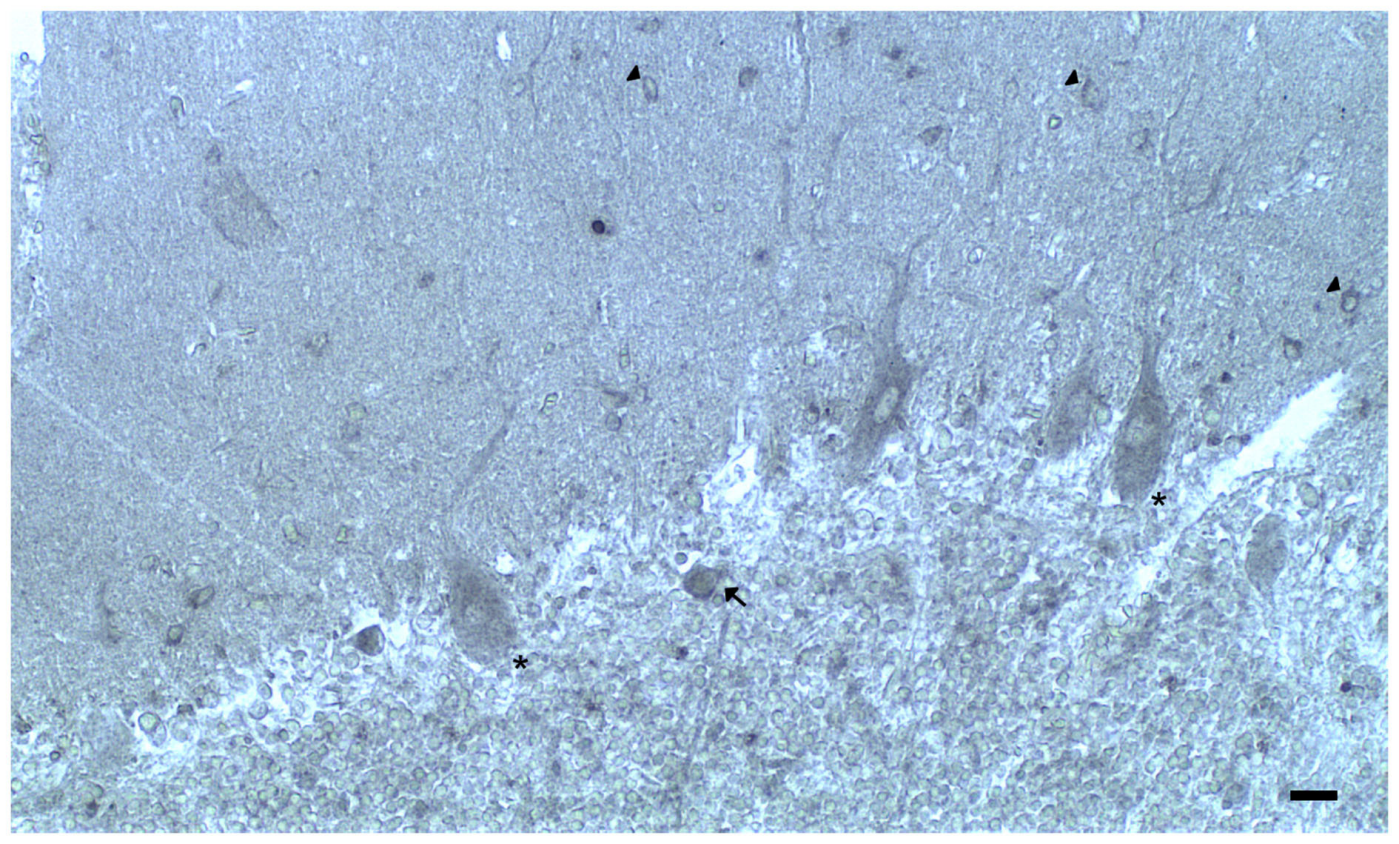
Dopamine receptor type 2 (DRD_2_) immunoreactivity is observable in neuronal bodies and processes in the layers of the cerebellar cortex. In the ML:DRD_2_ immunoreactive basket and stellate neuron cell bodies (arrows), DRD_2_ immunoreactive primary, secondary, and apical dendrites of Purkinje neurons, DRD_2_ immunoreactive Purkinje neuron cell bodies (*single asterisk*). In the granular layer, DRD_2_ immunoreactivity in space of Held; DRD_2_ immunoreactive Golgi neuron cell body (arrow) (Scale bar: 20 μm).

In the human cerebellum, immunohistochemical experiments revealed the presence of DAT immunoreactive fibers and neuronal cell bodies in lobules VII and IX (crus I and II, ansiform lobules, and tonsilla) and in the dentate nucleus (Table 1; Figures 1, 2; Flace, 2017, 2019b, 2020; Flace et al., 2018a, 2019b, 2020). There is a significant presence of DAT immunoreactive dendritic arborization of the Purkinje neurons in the molecular layer of the human cerebellar cortex (Table 1; Figure 1). Moreover, the DAT immunoreactivity has been detected in form of clusters in the neuropil among the space of Held, the sites of the cerebellar glomeruli (Table 1; Figure 1; Flace, 2017, 2019b, 2020; Flace et al., 2018a, 2019b, 2020).

**Figure 3:**
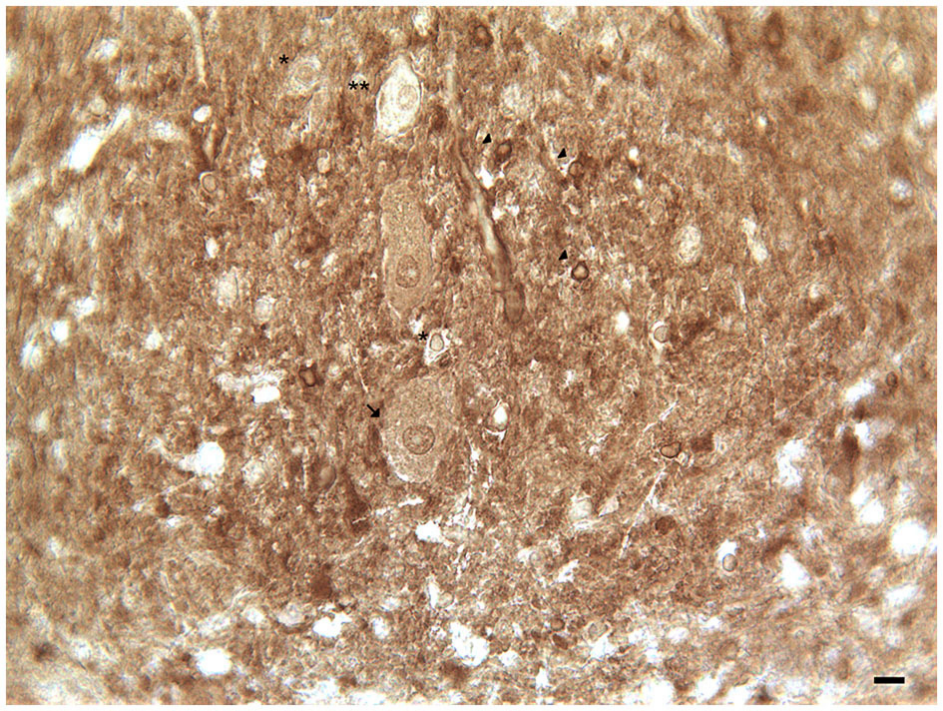
Dopamine transporter (DAT) immunoreactivity in the dentate nucleus. The dopamine transporter (DAT) immunoreactivity is detectable in the dentate nucleus gray substance and in the neighboring white substance. DAT immunoreactive small neuron cell bodies (*arrowheads*); DAT immunonegative small neuron cell body (*single asterisks*) DAT immunoreactive cell body of projective neuron type, central neuron (*arrow*); fine clusters of DAT immunoreactivity in the neuropil of the nucleus and in the neighboring withe substance (Scale bar: 15 μm).

In addition, the DAT immunoreactivity in the cell bodies of Purkinje neurons and of synarmotic neurons (Neuron of Landau) has been also detected (Table 1; Figure 1). This latter, one of the non-traditional large neuron granular layers was involved in corticocerebellar and in corticonuclear projective circuits (Flace et al., 2004, 2018a, 2019b, 2020; Ambrosi et al., 2007; Flace, 2017, 2019a,b, 2020). The DAT immunoreactivity in the cell bodies of few granules has been also detected (Flace et al., 2019a,b, 2020). Moreover, DAT immunoreactive nerve fibers variously oriented in the subcortical white substance, has been detected (Figure 1; Flace et al., 2019a,b, 2020). In the dentate nucleus, the DAT immunoreactivity in neuronal cell bodies and processes of different neuron types has been detected (Table 1; Figure 3); the small neuron type is involved in intrinsic circuits, the medium neuron type (Table 1; Figure 3) mainly involved in intrinsic and also in extrinsic circuits, and four different large neuron types, which include the central neuron, the border neuron, the intermediate asymmetrical neuron, and the intermediate fusiform neuron, involved in projective circuits of the dentate nucleus (data not showed; Chan-Palay, 1977; Maric, 2010; Ristanović et al., 2010; Flace et al., 2017, 2019b, 2020; Flace, 2018). Dopamine transporter immunoreactivity in neuronal cell bodies and processes of the perivascular neuron type has also been observed, a neuron type may be involved in regulatory mechanisms of blood–brain barrier (BBB) permeability and in volume transmission mechanisms (data not showed; Flace et al., 2004; Ambrosi et al., 2007; Flace, 2017, 2018, 2019b, 2020).

Furthermore, through different methods in the cerebellum of mammals, a wide distribution of the dopaminergic receptor subtypes (DRD_1_-DRD_5_) has been observed (Table 2; Camps et al., 1989; Cortés et al., 1989; Levant, 1998; Barili et al., 2000; Kiss et al., 2011; Flace et al., 2019b, 2020). A broad expression of all the dopaminergic receptor subtypes (DRD_1_-DRD_5_) has been demonstrated in the rodent and human cerebellum (Table 2; Martres et al., 1985; Camps et al., 1990; Mengod et al., 1992; Panagopoulos and Matsokis, 1994; Ricci et al., 1995a,b, 1996; Vessotskie et al., 1997; Levant, 1998; Barili et al., 2000; Khan et al., 2000; Hurley et al., 2003; Delis et al., 2004; Kim et al., 2009; Flace, 2017, 2018, 2019b; Flace et al., 2018a, 2019a,b, 2020).

**Table 2:** Distribution of the dopaminergic receptor subtypes in the mammalian cerebellum.

**Dopamine receptor subtypes**	**Molecular layer**	**Purkinje neuron layer**	**Granular layer**	**Deep cerebellar nuclei**
Dopamine Receptor D1 (DRD_1_)	- Stellate and Basket neuronal cell bodies and processes - Dendrites of the Purkinje neurons - Fine clusters of puncta (axon terminals)	- Purkinje neuron cell bodies	–	- Projective and associative neurons cell bodies and processes in the dentate nucleus
Dopamine Receptor D2 (DRD_2_)	- Stellate and Basket neuronal cell bodies and processes - Dendrites of the Purkinje neurons - Fine clusters of puncta (axon terminals)	- Purkinje neuron cell bodies	- Clusters in the neuropil in the glomeruli complex sites - In cell bodies and processes: a) Golgi neurons granules b) Lugaro neurons c) candelabrum neurons d) ellipsoidal neurons e) globular neurons f) perivascular neurons	- Fibers and puncta (axon terminals) - Projective and associative neurons cell bodies and processes in the dentate nucleus
Dopamine Receptor D3 (DRD_3_)	- Stellate and Basket neuronal cell bodies and processes - Dendrites of the Purkinje neurons - fine clusters of puncta (axon terminals)	- Purkinje neuron cell bodies	–	–
Dopamine Receptor D4 (DRD_4_)	–	–	Clusters in the neuropil in the glomeruli complex sites	–
Dopamine Receptor D5 (DRD_5_)	- Stellate and Basket neuronal cell bodies and processes - Dendrites of the Purkinje neurons - Fine clusters of puncta (axon terminals)	- Purkinje neuron cell bodies	–	–

In the three layers of the cerebellar cortex, the dopaminergic receptor subtypes present a different distribution pattern. In the molecular layer, immunoreactivity to DRD_2_, DRD_3_, and DRD_5_ receptors in the cell bodies and processes of stellate neurons, basket neurons, and in the dendritic arborizations of the Purkinje neurons has been detected (Table 2; Figure 2). Moreover, in the neuropil of the molecular layer, fine clusters of DRD_2_ immunoreactivity were detected (Table 2; Figure 2; Camps et al., 1990; Ricci et al., 1995b, 1996; Khan et al., 1998; Levant, 1998; Barili et al., 2000; Flace et al., 2018a, 2019a,b, 2020).

In the Purkinje neuron layer, DRD_1_, DRD_2_, DRD_3_, and DRD_5_ immunoreactive cell bodies of Purkinje neurons have been observed (Table 2; Figure 2; Camps et al., 1990; Bouthenet et al., 1991; Ricci et al., 1995a,b; Khan et al., 1998; Lazarov et al., 1998; Barili et al., 2000; Kim et al., 2009; Flace et al., 2018a, 2019a,b, 2020). In the granular layer, DRD_2_ immunoreactivity in the cell bodies and processes of granules, Golgi neurons (Table 2; Figure 2), and in different non-traditional large neuron types of the granular layer distributed in three zones has been detected (Flace et al., 2004; Flace, 2017, 2019b, 2020) such as the Lugaro neuron, candelabrum neuron, and perivascular neuron in the external zone of the layer, the triangular neuron in the intermediate zone, the ellipsoidal neuron, and the globular neuron in the internal zone has been detected (Table 2; data not showed; Flace et al., 2004; Ambrosi et al., 2007; Flace, 2017, 2019b, 2020). In addition, DRD_1_, DRD_2_, and DRD_4_ immunoreactive clusters in the neuropil of the granular layer have been found (Table 2; Figure 2), and DRD_2_ and DRD_5_ immunoreactivity in cell bodies of granules has been observed (Table 2; data not showed; Camps et al., 1990; Brouwer et al., 1992; Ricci et al., 1995a,b; Khan et al., 1998; Lazarov et al., 1998; Barili et al., 2000; Kim et al., 2009; Flace, 2017, 2019b; Flace et al., 2018a, 2019b, 2020). Furthermore, among immunonegative granules, the DRD_2_ immunoreactivity in form of clusters in the space of Held, the sites of the cerebellar glomeruli complex has been detected (Table 2; Figure 2; Flace et al., 2018a, 2019a,b, 2020).

**Figure 4:**
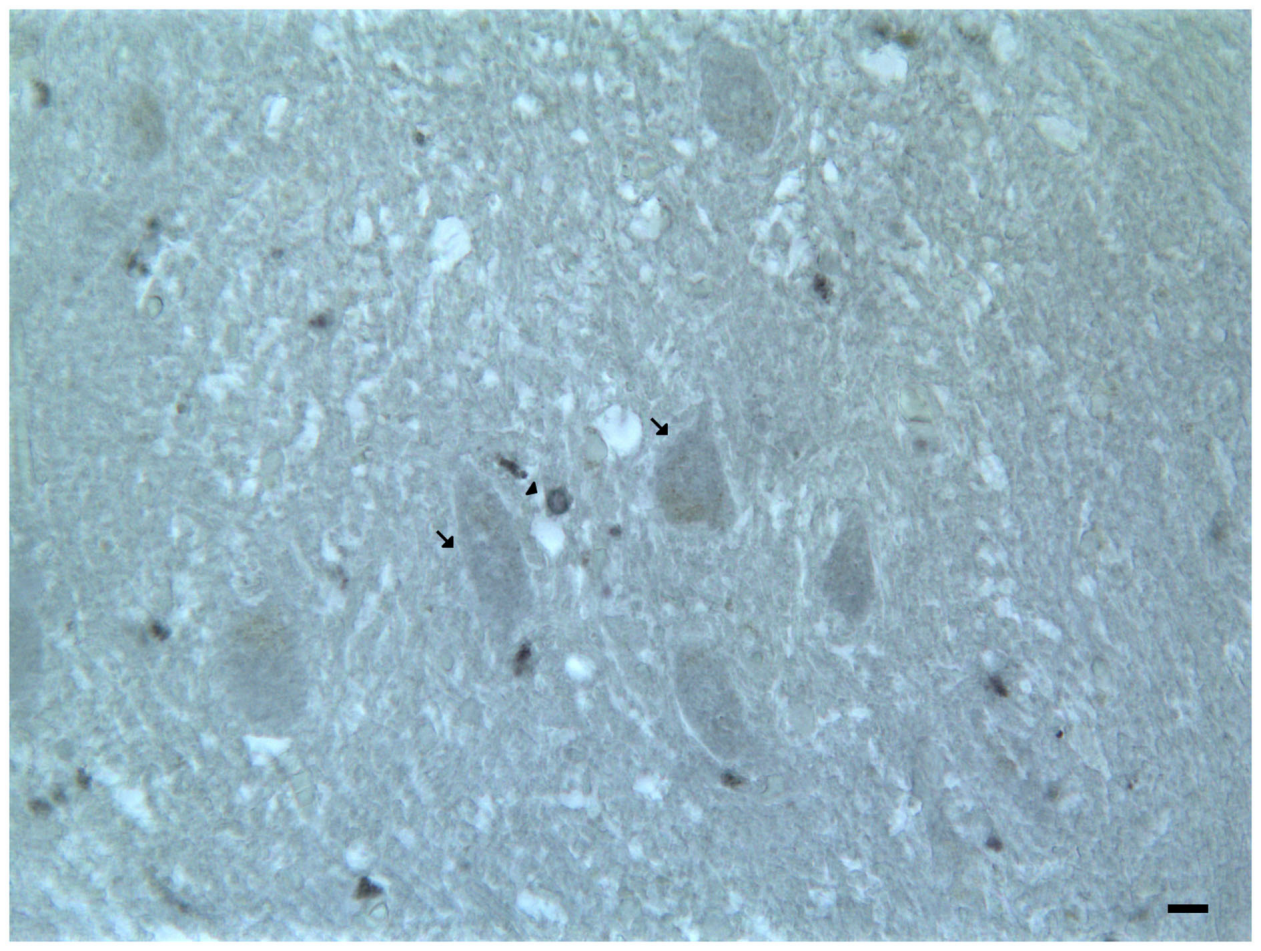
Dopamine receptor type 2 (DRD_2_) immunoreactivity in the dentate nucleus. The (DRD_2_) immunoreactivity is detectable in the dentate nucleus gray substance and in the neighboring withe substance; DRD_2_ immunoreactive small neuron cell bodies (arrowheads); DRD_2_ immunoreactive cell body of projective neuron type, central neuron (arrow); diffuse DAT immunoreactivity in the neuropil of the nucleus (Scale bar: 15 μm).

In the mouse and human dentate nucleus, the presence of DRD_1_ and DRD_2_ immunoreactive cell bodies and processes of different large projective neuron types and small associative neuron types has been demonstrated (Table 2; Figure 4); the DRD_2_ immunoreactivity has also been observed in form of fine clusters in the neuropil of the dentate nucleus (Table 2; Figure 4; Flace, 2017; Flace et al., 2018a, 2019a,b, 2020; Locke et al., 2018).

In chemical neuroanatomy studies carried out on the cerebellum by means of antisera directed against the TH, the rate-limiting enzyme of DA biosynthesis and the presence of numerous TH immunoreactive fibers in the various lobules and laminae of the cerebellar cortex as well as in the deep cerebellar nuclei have been demonstrated (Austin et al., 1992; Ikai et al., 1992; Takada et al., 1993, Nelson et al., 1997). In addition, regarding the TH immunoreactivity, it should be indicated that it is related to the presence of NA or DA, or both, since by carrying out a selective depletion of NA, most of the immunoreactivity is abolished (Fuxe, 1965; Hökfelt and Fuxe, 1969; Bloom et al., 1971). Moreover, using biochemical techniques, low levels of DA were found in the cerebellum (Carlsson, 1959; Glowinski and Iversen, 1966; Landis and Bloom, 1975). In addition, using antisera against DBH, the enzyme responsible for the biosynthesis of NA, highlights the presence of fibers in the cerebellum, which presented only partial similarity to those observed in studies using directed antisera against DA (Verney et al., 1988; Panagopoulos et al., 1991). Furthermore, more recent studies in the cerebellum of various mammals species and in other regions of the central nervous system (CNS), demonstrated which distribution patterns of TH immuroreactivity were mainly correlated to catecholaminergic and not electively to the dopaminergic neurotransmission but did not exclude it; (Fallon and Moore, 1978; Hökfelt et al., 1984; Asan, 1993; Takada et al., 1993; Nelson et al., 1997). On the contrary, studies that electively used antisera against DAT evaluate specifically the distribution patterns related to dopaminergic neurotransmission (Melchitzky and Lewis, 2000; Delis et al., 2008; Flace et al., 2018a, 2019b, 2020). In addition, the DAT immunohistochemial studies evidenced the presence of a specific subpopulation of dopaminergic neuronal cell bodies and processes in the cerebellum (Melchitzky and Lewis, 2000; Delis et al., 2008; Flace et al., 2018a, 2019b, 2020), which is in agreement with the studies on the distribution pattern of the dopaminergic receptors subtype in the cerebellar neuronal cell bodies and processes (Martres et al., 1985; Camps et al., 1990; Mengod et al., 1992; Ricci et al., 1996; Vessotskie et al., 1997; Levant, 1998; Barili et al., 2000; Khan et al., 2000; Delis et al., 2004; Kim et al., 2009; Flace et al., 2018a, 2019a,b, 2020).

In fact, the relationship of these data suggest the existence in the cerebellum of detailed dopaminergic neurotransmitter mechanisms. For example, in terms of the distribution pattern of the DAT immunoreactivity (Melchitzky and Lewis, 2000; Delis et al., 2008; Flace et al., 2018a, 2019b, 2020) and of the dopaminergic D1-like and D2-like subtype receptors immunoreactivity (Camps et al., 1990; Bouthenet et al., 1991; Ricci et al., 1995a,b; Khan et al., 1998; Lazarov et al., 1998; Barili et al., 2000; Kim et al., 2009; Flace et al., 2018a, 2019a,b, 2020), both were expressed in the Purkinje neurons cell bodies, dendritic arborizations, and axons, and this suggests the existence of a detailed cerebellar modulation by means of dopaminergic neurotransmission mechanisms in intrinsic and extrinsic cerebellar circuits.

## Physiological Aspects on the Dopaminergic Cerebellar System

In animal model studies, it has been indirectly demonstrated an active role of DA in the cerebellum; indeed, the administration of lacosamide and morphine in a hypoglycemic animal model decreased the cerebellar level of dopamine significantly (Guzman et al., 2014). In the cerebellum of albino rats, the long administration of morphine sulfate determines a decrease in the levels of DA and histopathological changes (Bekheet et al., 2010). Hypoxic conditions induced in the cerebellum of neonatal rats a decrease in the DA levels and a reduced expression of the dopaminergic subtype receptors DRD_1_ and DRD_2_; these decreases are in part reversed by the supplementation of glucose, oxygen, and adrenaline (Joseph et al., 2010). Moreover, in the cerebellum of rodents, high levels of DA have been involved in neuronal synaptic mechanisms characterized by DA release and uptake (Efthimiopoulos et al., 1991; Dethy et al., 1997). In addition, in mouse cerebellar slices, the presence of a high-affinity Na^+^-dependent DA uptake system has been demonstrated, and this has been characterized by a K^+^-induced, Ca^+2^-dependent dopamine release mechanism (Efthimiopoulos et al., 1991). Moreover, in several studies, it has been demonstrated in striatal medium spiny neurons a direct influence of DA in the mechanism of structural plasticity of dendritic spines (Yagishita et al., 2014). In the rat cerebellum, DA may influence in the Purkinje neurons the induction of RP a form of long-lasting synaptic plasticity at inhibitory synapses by means of the cAMP-regulated protein DARPP-32 highly expressed in Purkinje neurons and involved in dopaminergic neuronal synaptic signaling (Alder and Barbas, 1995; Kawaguchi and Hirano, 2002).

Moreover, in rat Purkinje neurons dendrites, a release of DA from vesicular extrasynaptic and postsynaptic sites resulted in dopaminergic receptors paracrine and autocrine activation (volume transmission), which produced a Depolarization-Induced Slow Current (DISC; Kim et al., 2009). Moreover, in pharmacological experiments a close functional relationship in dopaminergic Purkinje neurons between DA signaling and DISC has been demonstrated; in fact, it was blocked by dopaminergic receptor antagonist (e.g., clozapine, haloperidol, and eticlopride), VMAT_2_ inhibitors (reserpine and tetrabenazine), and dopamine reuptake inhibitors (e.g., rimcazole; Kim et al., 2009). Furthermore, it has been suggested in recent studies which TH immunoreactive Purkinje neurons and DRD_1_ immunoreactive large projective neuron types of the dentate nucleus may be involved in the modulation of cerebellar cognitive functions (Locke et al., 2018, 2020). The selective chemogenetic inhibition of the DRD_1_ immunoreactive neuron type of the dentate nucleus could be involved in the impairment of cognitive functions such as spatial navigation memory, working memory, and pre-pulse inhibition of the acoustic startle reflex (Locke et al., 2018). In mice, a selective reduction of TH immunoreactive cerebellar Purkinje neurons has been correlated to a specific impairment of cognitive functions, such as behavioral flexibility, response inhibition, social recognition memory (Locke et al., 2020).

From the analysis of these experimental physiological and pharmacological studies, a potential role of the neuronal dopaminergic system at the cerebellar level emerges, especially in the synaptic and extrasynaptic neurotransmission and neuromodulation mechanisms (Efthimiopoulos et al., 1991; Dethy et al., 1997; Kawaguchi and Hirano, 2002; Kim et al., 2009) and, in cognitive functions related to the cerebellar activity (Locke et al., 2018, 2020). Overall, they deserve further evaluation in order to better understand the relevance of the morphofunctional role played by the dopaminergic innervation in the cerebellum and their role in the behavioral functions of the cerebellum.

## Cerebellar–Midbrain Dopaminergic Pathways

In rodents, lesional and axonal tracing studies has been demonstrated that the cerebellar extrinsic dopaminergic fibers originate from the midbrain dopaminergic cell groups (A_8_-A_10_), which mainly consist of the ventral tegmental area (VTA) (A_10_) and to lesser extent by the retrorubral nucleus (A_8_) and the pars compacta of the substantia nigra (SNpc) (A_9_; Dahlström and Fuxe, 1964; Kizer et al., 1976; Chan-Palay, 1977; Oades and Halliday, 1987; Ikai et al., 1992; Melchitzky and Lewis, 2000; Kim et al., 2009).

In addition, in cat and in rat, a direct cerebellar influence on the midbrain dopaminergic nuclei (A_8_-A_10_) has been demonstrated. Fibers from the vermian cerebellar cortex and from the fastigial nucleus reach the ipsilateral VTA, whilst fibers from the interpositus and dentate nuclei reach the contralateral dorsal VTA and the medial and dorsal SNpc; moreover, 20% of the fibers had bilateral interconnections (**Figure 9**; Snider and Maiti, 1976).

In the rat cerebellum, using horseradish peroxidase (HRP) anterograde and retrograde transport methods, the efferents of the dentate and interpositus nuclei to the contralateral midbrain dopaminergic cell groups A_8_-A_10_ have been demonstrated (**Figure 9**; Perciavalle et al., 2013).

Electrical stimulation of cat cerebellar dentate nucleus influenced the dopaminergic activity of the ipsilateral SNpc, which in turn increased the release of [^3^H]-DA in the contralateral caudate nucleus and decreasing such release in the ipsilateral caudate nucleus. Moreover, the electrical stimulation of the fastigial nucleus increased only the release of [^3^H]-DA in the ipsilateral caudate nucleus (Nieoullon et al., 1978), and the electrical stimulation of the posterior interpositus nucleus increased the release of [^3^H]-DA in ipsilateral SNpc and in the contralateral caudate nucleus while decreasing the release [^3^H]-DA in the ipsilateral caudate nucleus (Nieoullon and Dusticier, 1980). In addition, the electrical stimulation of the mouse cerebellar dentate nucleus was elicited mainly in the contralateral nucleus accumbens (NAc), determining an asymmetrical and lateralized DA release (**Figure 9**; Holloway et al., 2019).

**Figure 5:**
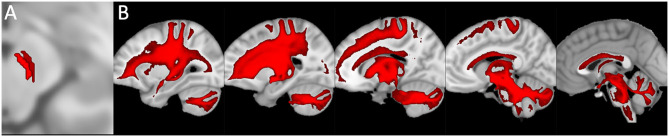
Structural connectivity of SNpc and VTA, including putative midbrain-cerebellar connectivity. Data were obtained from the 100-unrelated-subjects sample of the HCP repository (see Van Essen et al., 2013) Diffusion datasets were processed using a multi-shell, multi-tissue constrained spherical deconvolution (MSMT-CSD) algorithm (see Jeurissen et al., 2014). A number of 10,000 streamlines passing through the left SNpc (dark red) and VTA (light red) regions of interest (see Pauli et al., 2018) **(A)** was generated. Streamlines were mapped to structural scans, transformed to MNI152 standard space, binarized, and summed up to obtain tract maximum probability maps (MPMs). A threshold of 50% was applied to show only tracts overlapping in at least half of the sample **(B)**. Tractography was run on 30 high-quality 3T structural and diffusion data from the Human Connectome Project (HCP). Data were downloaded in a minimally pre-processed form and elaborated using the signal processing technique known as Constrained Spherical Deconvolution (CSD). Regions of interest (ROI) were delineated by means of multi-atlas automated segmentation: Substantia nigra (SN) and Ventro Tegmental Area (VTA) were resliced into subject space from Adcock's probabilistic atlas; dentate nucleus (both dorsal and ventral part) using the deep cerebellar nuclei atlas featured in SPM Anatomy Tract colors are attributed according to the spatial orientation of streamlines: superior-inferior (blue), anterior-posterior (green), and latero-lateral (red).

Moreover, in the last decades, the developments in neuroscience research of non-invasive and *in vivo* diffusion Magnetic Resonance Imaging and tractography have been increasingly used for the neuroanatomical reconstruction of putative white substance tracts or links of the human brain (Jeurissen et al., 2014; Cacciola et al., 2016a,b, 2017a,b, 2019). Although relatively few studies focused on the connectivity of midbrain nuclei, some of these reported structural connectivities between dopaminergic midbrain regions and the cerebellum (Bareš et al., 2015; Milardi et al., 2016; Cacciola et al., 2017a; Flace et al., 2017, 2018a,b, 2019a, 2020). An early work based on diffusion tensor imaging (DTI) and deterministic tractography aimed at the reconstruction of the median forebrain bundle (MFB), which represents the main white substance pathway connecting VTA and SNpc to the prefrontal cortex (PFC), found also a descending branch reaching to the cerebellum and in particular to the dentate nucleus through the superior cerebellar peduncle (SCP; Coenen et al., 2012). These findings have been replicated in a study by using more advanced signal modeling algorithms and different tracking strategies (Coenen et al., 2018). Nevertheless, results coming from diffusion imaging should be interpreted with care due to the well-known limitations of the tractographic approach, such as the inability to detect axons or synapses and, then, to rule out the precise termination of putative white substance tracts at a cellular level as well as to distinguish between direct or indirect connectivity patterns and passing-by fibers (Jbabdi and Johansen-Berg, 2011). In particular, the inherently low spatial resolution of diffusion-weighted MRI makes it difficult to distinguish between SNpc, SNpr, and VTA, as their precise boundaries are not readily identifiable on conventional MRI scans (Chowdhury et al., 2013; Trutti et al., 2019). In addition, these results may be affected by passing-by fibers from the dento-rubro-thalamic tract (DRTT), which lies in close proximity to midbrain dopaminergic structures, despite a recent study having suggested the potential dissociability of the cerebellar branch of MFB from DRTT (Hosp et al., 2019). In addition, in a human brain structural connectivity tractographic reconstruction of SNpc and VTA, we evidenced the existence of wide interconnections of the cerebellum with the SNpc and also with the VTA (Figure 5).

**Figure 6:**
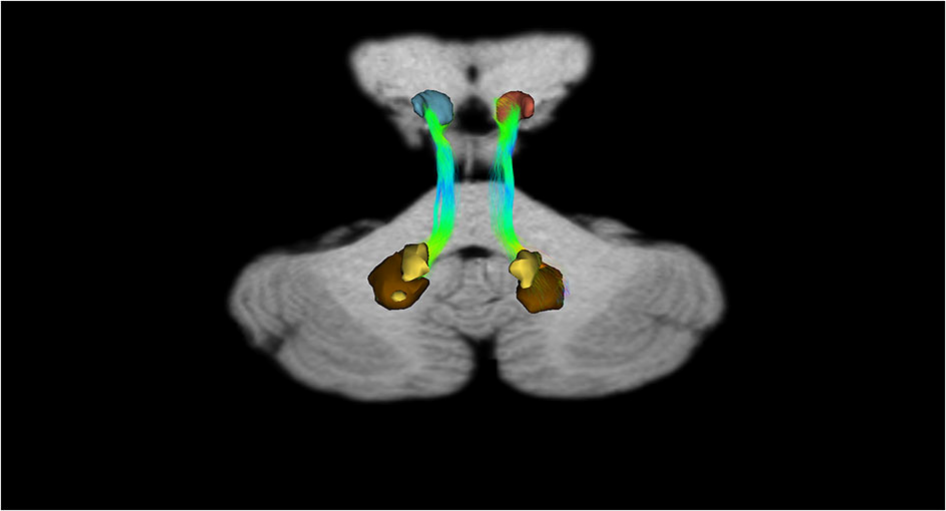
Dentate-nigral interconnections. Coronal view shows the interconnections between the right dentate nucleus and the ipsilateral SN, and the left dentate nucleus and the ipsilateral SN. The fibers exited the cerebellum via the right and left superior cerebellar peduncles.

**Figure 7:**
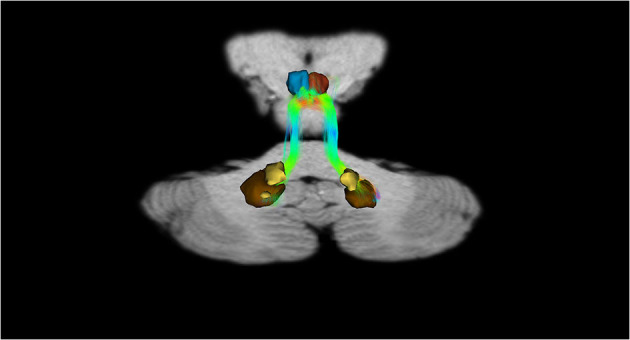
Dentate-VTA interconnections. Coronal view shows the interconnections between the right dentate nucleus and the ipsilateral and contralateral VTA, and the left dentate nucleus and the ipsilateral and contralateral VTA. The fibers exited the cerebellum via the right and left superior cerebellar peduncles.

**Figure 8:**
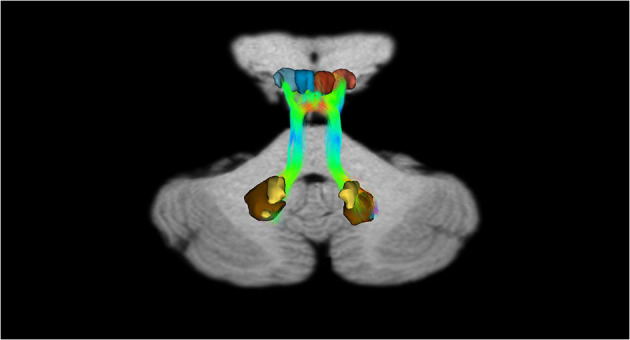
Dentate-SN and dentate-VTA interconnections. Coronal view shows the interconnections between the right dentate nucleus and left dentate nucleus to the ipsilateral SN, between the right dentate nucleus and left dentate nucleus to the ipsilateral and contralateral VTA. The fibers exited the cerebellum via the right and left superior cerebellar peduncles.

In addition, recently, by means of Constrained Spherical Deconvolution tractography (CSDt), Milardi et al. (2016) carried out a detailed analysis of direct links between the ventral and dorsal dentate nucleus and the ipsilateral SNpc (Figures 6, 8; Milardi et al., 2016). Subsequently, by means of CSDt, the existence of direct interconnections between the ventral and dorsal dentate nucleus and ipsilateral and contralateral VTA, predominantly characterized by an ipsilateral dentate-VTA links, has also been demonstrated (Figures 7, 8; Bareš et al., 2015; Milardi et al., 2016; Cacciola et al., 2017a; Flace et al., 2017, 2018a,b, 2019b, 2020).

**Figure 9:**
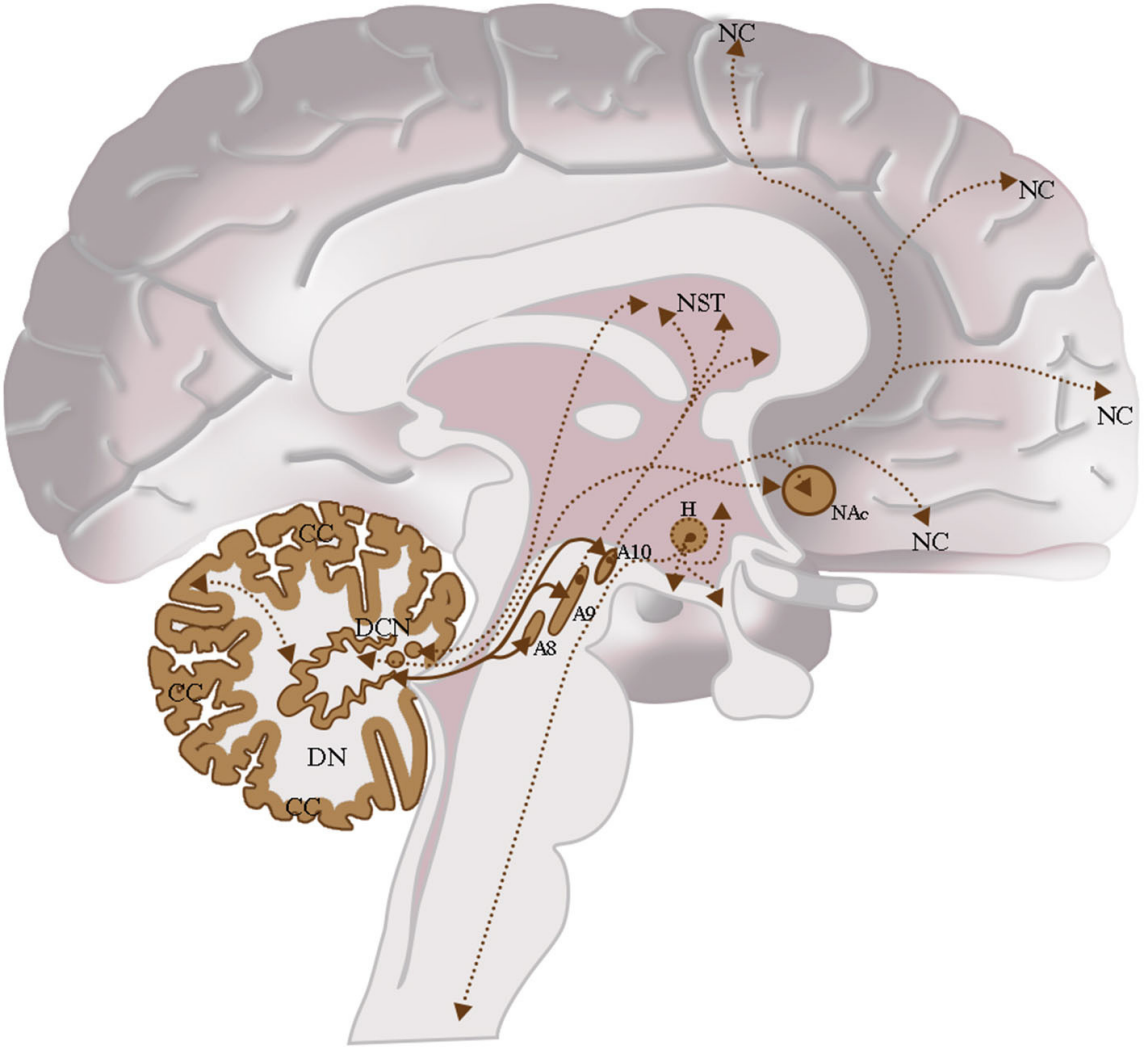
The dopaminergic CNS and their interconnections. Cerebellum: Cerebellar Cortex (CC), Deep Cerebellar Nuclei (DCN); Midbrain: Retrorubral Nucleus (A_8_), Substantia Nigra Pars Compacta (A_9_), Ventral Tegmental Area (A_10_); Hypothalamus (H); Nucleus Accumbens (NAc); Neostriatum (NST); Neocortex (NC). *Intrinsic cerebellar dopaminergic interconnections:* Between the dentate nucleus (DN) and the cerebellar cortex (CC); these interconnections are indicated in brown with the double arrow and the bold line. *Extrinsic cerebellar dopaminergic interconnections*: Between the dentate nucleus (DN) and the nuclei of the midbrain A_8_, A_9_, and A_10_; interconnections are indicated in brown with a double arrow and bold line. Between the dentate nucleus (DN) and the Neostriatum (NST); among others Deep Cerebellar Nuclei Nuclei (DCN) and the NST or the NAc. These interconnections are indicated in brown with the double arrow and the thin dashed line. *Other Dopaminergic Interconnections of the CNS*: Interconnection between the Ventral Tegmental Area (A_10_) and the NAc or between the Ventral Tegmental Area (A_10_) and the Neocortex (NC). These interconnections are indicated in brown with the double arrow and the thin dashed line.

The interconnection studies conducted with invasive methods in non-human mammals, and the analyses carried out in humans by means of tractographic neuroimaging methods highlight the presence of relevant interconnections of the cerebellum with the traditional dopaminergic areas of the brain. Moreover, this may likely suggest double direct functional DA interactions between the cerebellar dopaminergic system described in this review and the traditional DA cell groups system of the CNS (Figure 9; Björklund and Dunnett, 2007). In addition, these cerebellar-midbrain dopaminergic interconnections could represent part of the cerebellar projective circuits which allow the cerebellum to contribute to motor and cognitive functions (Koziol et al., 2014; Caligiore et al., 2017).

## The Role of the Dopaminergic Cerebellar System in Neurologic and Psychiatric Disorders

Though several studies suggested an involvement of the cerebellum in dopaminergic related neurologic and psychiatric disorders as PD (Jellinger, 1999, 2017; Lewis et al., 2013; Wu and Hallett, 2013), SCZ (Andreasen and Pierson, 2008; O'Hallaran et al., 2012; Parker et al., 2014), and ASD (O'Hallaran et al., 2012; Hampson and Blatt, 2015; Phillips et al., 2015), the precise role of the cerebellar dopaminergic system has not been fully characterized yet.

In this review, briefly, we analyzed some considerable experimental and clinical aspects of the cerebellum related to the dopaminergic system and its disorders.

Currently, only in few detailed studies has the direct involvement of a dopaminergic system at the cerebellar level in PD been analyzed. In a 6-hydroxydopamine (6-OHDA) animal model, increases in the DA level and its metabolites in the anterior cerebellum and as well as a decrease in the caudate-putamen have been detected (Kolasiewicz et al., 2012). In the cerebellum of PD patients, a reduced mRNA expression of TH and of some dopaminergic receptor subtypes (DRD_1_-DRD_3_) has been found (Hurley et al., 2003). In a human PD postmortem brain study it was shown that in the Purkinje neurons, a high expression of the calpain II (calpastatin), a calcium-dependent protease, resulted in overexpression in the dopaminergic neurons of SNpc (Mouatt-Prigent et al., 2000). PTEN-induced putative kinase 1 (PINK1) mutations related to the recessive genetic forms of parkinsonism, in the cerebellum of PD patients in Purkinje neuron and in several neuron types of dentate nuclei have been detected (Blackinton et al., 2007; Dodson and Guon, 2007).

The deposition of cerebellar α-synuclein (α-S) during PD remains unclear (Takahashi and Wakabayashi, 2001; Kingsbury et al., 2004). Indeed, some studies evidenced the presence of decreased or unchanged levels of α-S in the cerebellum (Tan et al., 2005; Westerlund et al., 2008), while others demonstrated a high mRNA expression of the α-S gene (SNCA) in the human cerebellum (Fuchs et al., 2008). Moreover, in the cerebellum of PD patients and of [A30P] transgenic mouse as well as in α-S in the molecular layer, the Bergmann glia (Mori et al., 2003; Piao et al., 2003), in the Purkinje neurons, in the space of Held of the granular layer, in the neuropil and in cell bodies and processes of different neuron types of the dentate nucleus has been found (Kahle et al., 2000; Mori et al., 2003). Furthermore, an α-S neuroprotective activity in cerebellar granules against neurotoxicity of 6-OHDA has been also demonstrated (Monti et al., 2007).

In rat cerebellum, high mRNA expression of clusterin/apolipoprotein J, a glycoprotein involved in the regulation of α-S deposition (Sasaki et al., 2002; Emamzadeh, 2017) in the Purkinje neurons as well as in the neurons of the fastigial and interpositus nuclei, has been detected (Pasinetti et al., 1994).

Currently, no studies are available on the direct involvement of the cerebellar dopaminergic system in SCZ and in autism ASD. However, in several studies, the presence of cerebellar abnormalities in SCZ and ASD patients has been demonstrated. In SCZ, patterns of atrophy in the cerebellar cortex of the vermis have been demonstrated (Weinberger et al., 1980; Reyes and Gordon, 1981; Heath et al., 1982; Snider, 1982; Martin and Albers, 1995). In addition, reduced cerebellar cortical volumes (Laidi et al., 2015), a decreased cerebellar gray substance of Crus I and II ansiform lobules (Kühn et al., 2012), and a reduction in the gyrification index in the cerebellar vermis have also been observed (Schmitt et al., 2011). Moreover, in the cerebellum of SCZ patients, in a microscopical analysis, a loss or a reduced cell size of the Purkinje neurons has been revealed (Stevens, 1982; Tran et al., 1998). Furthermore, a reduced cerebellar expression of the Sp transcription factors and DRD_2_, both related to negative symptoms of SCZ, has been found (Pinacho et al., 2013).

In ASD morphological studies, in the cerebellar hemispheres a reduction of the number of the Purkinje neurons related to a reduction of the Nissl staining has been demonstrated (Bauman and Kemper, 1985; Kemper and Bauman, 1993). Furthermore, in the cerebellum of ASD patients, we also found a reduced Purkinje neuron density (Whitney et al., 2008; Skefos et al., 2014) together with the decreased cell body size of the Purkinje neuron (Fatemi et al., 2002).

Moreover, studies suggested that SCZ and ASD symptoms, in part, may be derived from abnormalities of cerebro-cerebellar interconnections (Andreasen et al., 1998; Strick et al., 2009; Mosconi et al., 2015).

Furthermore, electrical stimulations of the Purkinje neuron layer and of the dentate nucleus evokes a long-lasting increase of DA efflux in the PFC, and this suggests a possible disconnection between the Purkinje neurons and neuronal population of the dentate nucleus, which in turn can lead to aberrant DA signaling in the PFC and to abnormal behavior related to symptoms of SCZ and ASD (Mittleman et al., 2008; Rogers et al., 2013).

Therefore, the cerebellum and its dopaminergic innervation and their interconnections to the other midbrain dopaminergic areas suggested a direct cerebellar involvement in the PD pathophysiological mechanisms (Lewis et al., 2013; Wu and Hallett, 2013; Yoo et al., 2019). Furthermore, the relevant role of the cerebellum is also strongly indicated in psychiatric disorders such as SCZ and ASD characterized by a significant dysregulation of the dopaminergic system (Andreasen et al., 1998; Strick et al., 2009; Mosconi et al., 2015).

## The Role of the Dopaminergic Cerebellar System in the Treatment of Neurologic and Psychiatric Dopamine-Related Disorders

Taken together, the data evidenced in the present review, suggested the existence of a cerebellar dopaminergic neuronal system, which can be the target for pharmacological, non-pharmacological, or combined therapeutic treatments (Miterko et al., 2019); here, we will briefly review some of the therapeutic aspects on the cerebellar dopaminergic system in PD, SCZ, and ASD.

In PD, neuroimaging studies have demonstrated L-DOPA administration resulted involved in asymmetrical effects in motor brain regions, highlighting differences in cerebellar activity (Martinu et al., 2014). In PD patients, an increased putamen-cerebellar activity after abstention of L-DOPA administration has been proven, suggesting a role for the cerebellum in compensatory mechanisms (Simioni et al., 2015).

In SCZ antipsychotic treatments, the cerebellum may also represent part of the pharmacologic target. In rat cerebellum, the atypical antipsychotic blonaserin and the anxiolytic buspirone engage extensively in dopamine receptor DRD_3_ (Baba et al., 2015; Di Ciano et al., 2017); indeed, in the cerebellum an extensive distribution of the dopamine receptor DRD_3_ has been demonstrated (Barili et al., 2000; Kim et al., 2009). Furthermore, in genomic DNA isolated from the cerebellum, the atypical antipsychotic agent olanzapine increased methylation of genes related to the dopaminergic system, such as DRD_5_, DOPA decarboxylase (DDC8), and VMAT_2_ (SCL18A2/VMAT2; Melka et al., 2013).

The cerebellum is extensively interconnected to the other brain regions involved in motor, cognitive, and affective functions (Milardi et al., 2016; Cacciola et al., 2017a, 2019; Caligiore et al., 2017; Bostan and Strick, 2018; Flace et al., 2018b). Although, these cerebellar interconnections have not yet been fully characterized, in studies, it has been demonstrated that the cerebellum may represent the ideal target of non-invasive brain stimulation therapies such as electrical or magnetic stimulations applied in therapies for neurological and psychiatric disorders (van Dun et al., 2017; Miterko et al., 2019; Quartarone et al., 2020). In PD patients, bilateral cerebellar repetitive Transcranial Magnetic Stimulation (rTMS) induced persistent clinical beneficial effects, reducing peak-dose L-DOPA-induced dyskinesia (Koch, 2010).

In healthy subjects, cerebellar vermal theta burst stimulation (TBS) produced downstream changes in neuronal activity in the frontal cortex (Schutter et al., 2003), and pharmacological treatment-resistant SCZ patients can improved cognitive functions (Demirtas-Tatlidede et al., 2010). The rTMS In ASD has been used to study excitatory/inhibitory imbalance (Uzunova et al., 2016) and can represent an innovative therapeutic approach for reducing some of the core and associated ASD symptoms (Oberman et al., 2016).

## Discussion and Conclusion

The present review extensively evidenced the available morphological, chemical, and functional data on the existence of a cerebellar dopaminergic system in mammals including humans, which consist of extrinsic fibers which originate mainly from the midbrain cerebellar dopaminergic nuclei (A_8_-A_10_; Ikai et al., 1992; Nelson et al., 1997) and of intrinsic dopaminergic neuronal subpopulations mainly composed of cortico-cerebellar projective neuron types, such as the Purkinje neuron and the synarmotic neuron, and by different cerebello-nuclear neuron types (Nelson et al., 1997; Delis et al., 2008; Flace, 2017; Flace et al., 2018a, 2019b).

In addition, this review evidenced the presence of direct dentate-SNpc and dentate-VTA interconnections (Milardi et al., 2016; Flace et al., 2017, 2018a, 2019b, 2020), which may play a relevant modulatory role in DA release at the PFC (Mittleman et al., 2008; Rogers et al., 2013) and highlight the possible involvement of dopaminergic cerebellar circuits in dopaminergic related disorders such as PD (Wu and Hallett, 2013; Flace et al., 2018a, 2019b, 2020), SCZ (Martin and Albers, 1995; Mittleman et al., 2008; Rogers et al., 2013; Parker et al., 2014), and ASD (Kemper and Bauman, 1993; Mittleman et al., 2008; Rogers et al., 2013).

Finally, we suggest that the cerebellar dopaminergic system and its interconnections may represent an ideal candidate for innovative non-invasive treatments such as electrical or magnetic stimulations in neurological and psychiatric disorders (Demirtas-Tatlidede et al., 2010; Koch, 2010; Oberman et al., 2016; Miterko et al., 2019; Quartarone et al., 2020). These innovative therapeutic objectives constitute relevant elements of study and we hope that they can be achieved in a relatively short time.

## Author Contributions

PF designed the study, performed the experiments and the analysis of the experimental data, and participated in the writing of the manuscript. PL and DG shared the study project and participated in the writing of the manuscript. GB, ABi, SBe, JB, SBi, and ABr participated in the writing of the manuscript. GG, MG, and GA performed the analysis of the experimental data, participated in the writing of the manuscript. All authors contributed to the article and approved the submitted version.

## Conflict of Interest

The authors declare that the research was conducted in the absence of any commercial or financial relationships that could be construed as a potential conflict of interest.

## Publisher's Note

All claims expressed in this article are solely those of the authors and do not necessarily represent those of their affiliated organizations, or those of the publisher, the editors and the reviewers. Any product that may be evaluated in this article, or claim that may be made by its manufacturer, is not guaranteed or endorsed by the publisher.
